# Supplementary material for: The Small RNA Universe of Capitella teleta
Source: Front Mol Biosci. 2022 Feb 25;9:802814. doi: 10.3389/fmolb.2022.802814 (PMC8915122; doi:10.3389/fmolb.2022.802814)
Supplement: Supplementary file 1 [file DataSheet1.ZIP › Supplement/known/CAPTEscaffold_933_30853.pdf]

Provisional ID	:	CAPTEscaffold_933_30853
Score total	:	500715.4
Score for star read(s)	:	3.9
Score for read counts	:	500705.5
Score for mfe	:	1.4
Score for randfold	:	1.6
Score for cons. seed	:	3
Total read count	:	982124
Mature read count	:	981576
Loop read count	:	140
Star read count	:	408

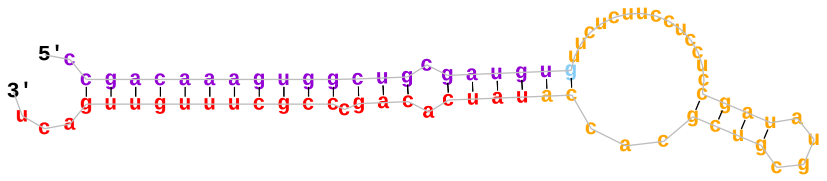

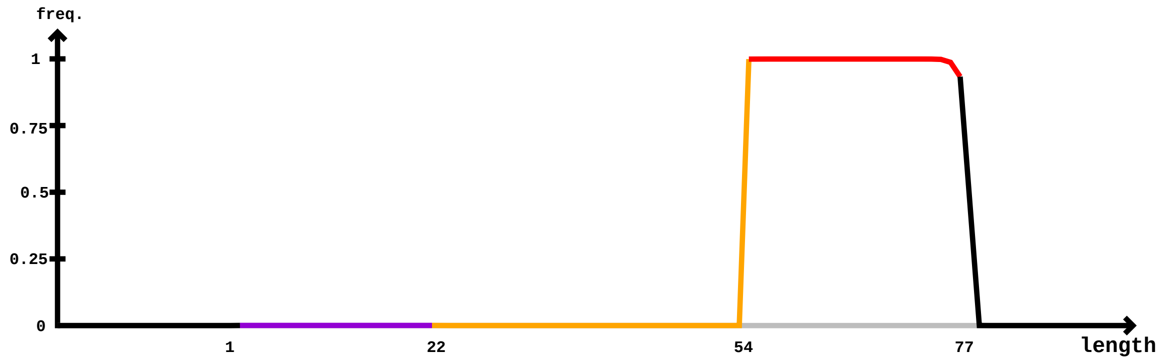

Star **Mature**

```
5'- gaucaaucauggaggaaccgacaaaguggcugcgauguguucucuuccuccuccgauaugcgucgcaccauaucacagcccgcuuuguugacucugccgugcaauacucu  -3'  obs
    gaucaaucauggaggaaccgacaaaguggcugcgaugugguucucuuccuccuccgauaugcgucgcaccauaucacagcccgcuuuguugacucugccgugcaauacucu       exp
    gaucaaucauggaggaaccgacaaaguggcugcgauguguucucuuccuccuccgauaugcgucgcaccauaucacagcccgcuuuguugacucugccgugcaauacucu       known
    .......(((((.(((..((((((((((((((.((((((..............((((....))))...)))))).))).)))))))))))..))).)))))........   reads  mm  sample
    ................accgacaaaguggcugcgaugu........................................................................   24     0   seq
    .................ccgacaaaguggcugcgaug.........................................................................   2      0   seq
    .................ccgCcaaaguggcugcgaugu........................................................................   1      1   seq
    .................ccgacGaaguggcugcgaugu........................................................................   1      1   seq
    .................ccgacaaaguggcugcgaugu........................................................................   303    0   seq
    .................Acgacaaaguggcugcgaugu........................................................................   1      1   seq
    .................ccgacaaaguAgcugcgaugu........................................................................   1      1   seq
    .................ccgacaaaguggcugcgaugA........................................................................   1      1   seq
    .................ccgacaaagAggcugcgaugu........................................................................   1      1   seq
    .................ccgacaaaguggcugcgauguU.......................................................................   1      1   seq
    .................Ucgacaaaguggcugcgaugug.......................................................................   1      1   seq
    .................ccgacaaaguggcugcgaugug.......................................................................   67     0   seq
    .................ccgacaaaguggcugcgaugugG......................................................................   1      1   seq
    .................ccgacaaaguggcugcgaugugu......................................................................   3      0   seq
    .................ccgacaaaguggcugcgauguguucucuu................................................................   1      0   seq
    .......................................uucucuuccuccuccgauaugcgucg.............................................   1      0   seq
    .......................................uucucuuccuccuccgauaugcgucgcacc.........................................   19     0   seq
    .......................................uucucuuccuccuccgauaugcgucgcacU.........................................   1      1   seq
    .......................................uucucuuccuccuccgauaugcgucgcaccU........................................   1      1   seq
    .......................................uucucuuccAccuccgauaugcgucgcacca........................................   1      1   seq
    .......................................uucucuuccuccAccgauaugcgucgcacca........................................   1      1   seq
    .......................................uucucuuccuccuccgauaugcgucgcacca........................................   114    0   seq
    ...................................................uccgauaugcgucgcacca........................................   2      0   seq
    ....................................................................Gauaucacagcccgcuuuguugacu.................   1      1   seq
    ....................................................................cCuaucacagcccgcuuuguugacu.................   1      1   seq
    .....................................................................Cuaucacagcccgcuuuguuga...................   1      1   seq
    .....................................................................Cuaucacagcccgcuuuguugac..................   3      1   seq
    .....................................................................auaucacagcccgcuuuguugacu.................   3      0   seq
    .....................................................................Guaucacagcccgcuuuguugacu.................   1      1   seq
    .....................................................................Cuaucacagcccgcuuuguugacu.................   37     1   seq
    ......................................................................uaucacagcccgcuuugu......................   40     0   seq
    ......................................................................uaucacagcccgcCuuguu.....................   1      1   seq
```

Star Mature

```
gaucaaucauggaggaaccgacaaaguggcugcgauguguucucuuccuccuccgauaugcgucgcaccauaucacagcccgcuuuguugacucugccgugcaauacucu
......................................................................uaucacagcccgcuuuguu.....................  116    0  seq
......................................................................uaucacagcccgcuCuguu.....................  1      1  seq
......................................................................Aaucacagcccgcuuuguug....................  4      1  seq
......................................................................Gaucacagcccgcuuuguug....................  3      1  seq
......................................................................uaucacagcccgUuuuguug....................  1      1  seq
......................................................................uaucacaCcccgcuuuguug....................  1      1  seq
......................................................................uaucacagcccgcuuuguuA....................  3      1  seq
......................................................................uaucGcagcccgcuuuguug....................  1      1  seq
......................................................................uaucacagcccgcuuuguuU....................  2      1  seq
......................................................................uUucacagcccgcuuuguug....................  1      1  seq
......................................................................uaAcacagcccgcuuuguug....................  3      1  seq
......................................................................uaucacagcccgcuuuguug....................  1113   0  seq
......................................................................uaucacagcAcgcuuuguug....................  1      1  seq
......................................................................uaucaAagcccgcuuuguug....................  1      1  seq
......................................................................uaucacagcccgcuuAguug....................  1      1  seq
......................................................................uaucacagcccgcuCuguuga...................  1      1  seq
......................................................................uaucacagcccgcuuuguuAa...................  9      1  seq
......................................................................uaucacagcAcgcuuuguuga...................  4      1  seq
......................................................................uaucacagcccgcuuugCuga...................  4      1  seq
......................................................................uaucGcagcccgcuuuguuga...................  1      1  seq
......................................................................uauAacagcccgcuuuguuga...................  5      1  seq
......................................................................uaucacagcccUcuuuguuga...................  1      1  seq
......................................................................uaucacagcccAcuuuguuga...................  2      1  seq
......................................................................Naucacagcccgcuuuguuga...................  2      1  seq
......................................................................uaucacagcccgUuuuguuga...................  1      1  seq
......................................................................uaucacagcccgcuuAguuga...................  5      1  seq
......................................................................uaucacagcccgAuuuguuga...................  3      1  seq
......................................................................Aaucacagcccgcuuuguuga...................  31     1  seq
......................................................................uaucacagcccgcuuugGuga...................  2      1  seq
......................................................................uaucacagcGcgcuuuguuga...................  1      1  seq
......................................................................uaucacagcccgcuuuCuuga...................  1      1  seq
......................................................................uaucacUgcccgcuuuguuga...................  1      1  seq
......................................................................uaucacagcccgcuuuguugU...................  5      1  seq
......................................................................uUucacagcccgcuuuguuga...................  1      1  seq
......................................................................uaucaAagcccgcuuuguuga...................  1      1  seq
......................................................................Gaucacagcccgcuuuguuga...................  4      1  seq
......................................................................uaucacagcccgcGuuguuga...................  1      1  seq
......................................................................uaucacagccAgcuuuguuga...................  3      1  seq
......................................................................uaucacagAccgcuuuguuga...................  1      1  seq
......................................................................uaAcacagcccgcuuuguuga...................  8      1  seq
......................................................................uaucacagccUgcuuuguuga...................  9      1  seq
......................................................................uaucacGgcccgcuuuguuga...................  2      1  seq
......................................................................uaucacagcccgcuAuguuga...................  1      1  seq
......................................................................uaCcacagcccgcuuuguuga...................  1      1  seq
......................................................................uaucacagcccgcCuuguuga...................  6      1  seq
......................................................................uaucacagcccgcuuuguCga...................  3      1  seq
......................................................................uauUacagcccgcuuuguuga...................  3      1  seq
......................................................................uGucacagcccgcuuuguuga...................  1      1  seq
......................................................................uaucacagcccCcuuuguuga...................  1      1  seq
......................................................................uaucacagcccgcuuugAuga...................  6      1  seq
......................................................................Caucacagcccgcuuuguuga...................  1      1  seq
......................................................................uaucacagUccgcuuuguuga...................  2      1  seq
......................................................................uaucacagcccgcuuuguuga...................  10628  0  seq
......................................................................uaucacagcccgcuuuguugG...................  37     1  seq
......................................................................uaucacagcccgcAuuguuga...................  1      1  seq
......................................................................uaucacagcUcgcuuuguuga...................  4      1  seq
......................................................................uaucacagcccgcuuuUuuga...................  1      1  seq
......................................................................uaucacagcAcgcuuuguugac..................  34     1  seq
......................................................................uaucacagccAgcuuuguugac..................  30     1  seq
......................................................................uaucacagcccUcuuuguugac..................  3      1  seq
......................................................................uaucacagGccgcuuuguugac..................  3      1  seq
......................................................................uauUacagcccgcuuuguugac..................  26     1  seq
......................................................................uaucacagUccgcuuuguugac..................  16     1  seq
......................................................................uGucacagcccgcuuuguugac..................  9      1  seq
......................................................................uauAacagcccgcuuuguugac..................  18     1  seq
......................................................................uaucacaAcccgcuuuguugac..................  8      1  seq
......................................................................uaucacagcccgcuAuguugac..................  25     1  seq
......................................................................uaucacagcccgcuCuguugac..................  4      1  seq
......................................................................uaucacagcccgcuuuguCgac..................  6      1  seq
......................................................................uaucaGagcccgcuuuguugac..................  2      1  seq
```

Star **Mature**

```
gaucaaucauggaggaaccgacaaaguggcugcgauguguucucuuccuccuccgauaugcgucgcaccauaucacagcccgcuuuguugacucugccgugcaauacucu
......................................................................uaucacagcccgcuuAguugac..................   37      1   seq
......................................................................uaCcacagcccgcuuuguugac..................   10      1   seq
......................................................................uaucacagcccgcAuuguugac..................   5       1   seq
......................................................................uaucacagcccgcuuuguuAac..................   12      1   seq
......................................................................uaucacaNcccgcuuuguugac..................   1       1   seq
......................................................................uaucacagcccgcuuuguugac..................   52022   0   seq
......................................................................Aaucacagcccgcuuuguugac..................   184     1   seq
......................................................................uaucacagcccgcuuuCuugac..................   5       1   seq
......................................................................uaucacagcccgcuuuguuUac..................   2       1   seq
......................................................................Naucacagcccgcuuuguugac..................   13      1   seq
......................................................................uaucacaCcccgcuuuguugac..................   4       1   seq
......................................................................uaucacagcccAcuuuguugac..................   7       1   seq
......................................................................uaucUcagcccgcuuuguugac..................   7       1   seq
......................................................................uaucacagcccgcuuuguugUc..................   10      1   seq
......................................................................uaucacagcccgcuuCguugac..................   3       1   seq
......................................................................uaucacagccUgcuuuguugac..................   36      1   seq
......................................................................uaucacagcccgcuuuAuugac..................   1       1   seq
......................................................................uaucacaUcccgcuuuguugac..................   3       1   seq
......................................................................uaucacagcccgUuuuguugac..................   2       1   seq
......................................................................uaucacagcccgcuGuguugac..................   1       1   seq
......................................................................uaucacagcccgcCuuguugac..................   30      1   seq
......................................................................uaucCcagcccgcuuuguugac..................   3       1   seq
......................................................................uaucacagcccgcuuuUuugac..................   2       1   seq
......................................................................uaAcacagcccgcuuuguugac..................   60      1   seq
......................................................................uaucacagcccgcuuuguugCc..................   1       1   seq
......................................................................uaucacagcccgcuuuguugaA..................   12      1   seq
......................................................................uaucacagcccgcuuugCugac..................   8       1   seq
......................................................................uaucacagAccgcuuuguugac..................   14      1   seq
......................................................................uaucGcagcccgcuuuguugac..................   14      1   seq
......................................................................uaucacagcccgAuuuguugac..................   22      1   seq
......................................................................uaucacCgcccgcuuuguugac..................   5       1   seq
......................................................................uauGacagcccgcuuuguugac..................   1       1   seq
......................................................................uaucacGgcccgcuuuguugac..................   12      1   seq
......................................................................Caucacagcccgcuuuguugac..................   5       1   seq
......................................................................uaucacagcccgcuuuguAgac..................   20      1   seq
......................................................................uaucacagcccgcuuuguugaU..................   25      1   seq
......................................................................uaucacagcccgcuuuguugaG..................   6       1   seq
......................................................................uaucaAagcccgcuuuguugac..................   14      1   seq
......................................................................uaucacagcccgcuuuguuCac..................   3       1   seq
......................................................................uUucacagcccgcuuuguugac..................   2       1   seq
......................................................................Gaucacagcccgcuuuguugac..................   23      1   seq
......................................................................uaucaUagcccgcuuuguugac..................   5       1   seq
......................................................................uaucacagcUcgcuuuguugac..................   23      1   seq
......................................................................uaucacagcGcgcuuuguugac..................   3       1   seq
......................................................................uNucacagcccgcuuuguugac..................   3       1   seq
......................................................................uaucacagcccgcuuugAugac..................   12      1   seq
......................................................................uaucacagcccgcuuuguugGc..................   11      1   seq
......................................................................uaGcacagcccgcuuuguugac..................   1       1   seq
......................................................................Aaucacagcccgcuuuguugacu.................   2859    1   seq
......................................................................uaucacagcccgUuuuguugacu.................   114     1   seq
......................................................................uaucacagccAgcuuuguugacu.................   516     1   seq
......................................................................uaucGcagcccgcuuuguugacu.................   225     1   seq
......................................................................uaucacagcccgcuuuguugaGu.................   53      1   seq
......................................................................uaucaNagcccgcuuuguugacu.................   6       1   seq
......................................................................Gaucacagcccgcuuuguugacu.................   460     1   seq
......................................................................uaucacagcccgcuuuguCgacu.................   119     1   seq
......................................................................uCucacagcccgcuuuguugacu.................   3       1   seq
......................................................................uaucacCgcccgcuuuguugacu.................   31      1   seq
......................................................................uaucacaCcccgcuuuguugacu.................   49      1   seq
......................................................................uaucacagcccgcCuuguugacu.................   739     1   seq
......................................................................uaucacagUccgcuuuguugacu.................   109     1   seq
......................................................................uaucacagcccgcuuuguugUcu.................   84      1   seq
......................................................................uaucacagcccgcuuuAuugacu.................   117     1   seq
......................................................................uaucaAagcccgcuuuguugacu.................   192     1   seq
......................................................................uGucacagcccgcuuuguugacu.................   111     1   seq
......................................................................uaucacagcccgcuuuguugCcu.................   19      1   seq
......................................................................uaucacagcccgcuuGguugacu.................   14      1   seq
......................................................................uaucacagcccgcuuuguugacu.................   896808  0   seq
......................................................................uaucacagcccgcuuuguugacN.................   6       1   seq
......................................................................uaucaUagcccgcuuuguugacu.................   117     1   seq
```

```
                      Star                                                  Mature
gaucaaucauggaggaaccgacaaaguggcugcgauguguucucuucccuccuccgauaugcgucgcaccauaucacagcccgcuuuguugacucugccgugcaauacucu
.......................................................................uaucacagcccgcuuuCuugacu.................   72     1   seq
.......................................................................uaucacagcccgcuuuguugacA.................   1109   1   seq
.......................................................................uaucacagAccgcuuuguugacu.................   249    1   seq
.......................................................................uaucacagcGcgcuuuguugacu.................   64     1   seq
.......................................................................uaucUcagcccgcuuuguugacu.................   90     1   seq
.......................................................................uaucacagcccgcuuuguugacC.................   186    1   seq
.......................................................................uaucacagcccgcuuuguAgacu.................   324    1   seq
.......................................................................uaucacaUcccgcuuuguugacu.................   60     1   seq
.......................................................................uaCcacagcccgcuuuguugacu.................   88     1   seq
.......................................................................uaucacagcccgcuuuguuCacu.................   51     1   seq
.......................................................................uaucacagccUgcuuuguugacu.................   774    1   seq
.......................................................................uaucacagcccgcuuCguugacu.................   98     1   seq
.......................................................................uaucaGagcccgcuuuguugacu.................   48     1   seq
.......................................................................uaucacGgcccgcuuuguugacu.................   225    1   seq
.......................................................................uaucacagcccgcuuuguugGcu.................   126    1   seq
.......................................................................uaucacagcccgcuuuguugaUu.................   128    1   seq
.......................................................................uaucacagcccgcuuuUuugacu.................   79     1   seq
.......................................................................uaucacagcccgcuuuguuUacu.................   36     1   seq
.......................................................................uaucacagcccgcAuuguugacu.................   90     1   seq
.......................................................................uaucacagcccgcuuuguugaAu.................   175    1   seq
.......................................................................uaucacagcccAcuuuguugacu.................   137    1   seq
.......................................................................uaucacagcAcgcuuuguugacu.................   440    1   seq
.......................................................................uaNcacagcccgcuuuguugacu.................   4      1   seq
.......................................................................uaucacagcccgcuuugCugacu.................   96     1   seq
.......................................................................uaucacagcccUcuuuguugacu.................   69     1   seq
.......................................................................uauUacagcccgcuuuguugacu.................   361    1   seq
.......................................................................uauAacagcccgcuuuguugacu.................   216    1   seq
.......................................................................uaucacagcccgcuuuguGgacu.................   34     1   seq
.......................................................................Naucacagcccgcuuuguugacu.................   229    1   seq
.......................................................................uaucacagccGgcuuuguugacu.................   70     1   seq
.......................................................................uaucacagcccgcuuugGugacu.................   31     1   seq
.......................................................................uaucacagcUcgcuuuguugacu.................   545    1   seq
.......................................................................uauGacagcccgcuuuguugacu.................   37     1   seq
.......................................................................uaucacagcccgAuuuguugacu.................   345    1   seq
.......................................................................uaucacagcccgcuuuguuAacu.................   360    1   seq
.......................................................................uaucacagcccgcuCuguugacu.................   83     1   seq
.......................................................................uaucacagcccgcuuAguugacu.................   676    1   seq
.......................................................................uaucacagGccgcuuuguugacu.................   39     1   seq
.......................................................................uaucacagcccgGuuuguugacu.................   62     1   seq
.......................................................................uaucacagcccgcuuuguugaNu.................   2      1   seq
.......................................................................uaucacagcccCcuuuguugacu.................   48     1   seq
.......................................................................uaucacaAcccgcuuuguugacu.................   67     1   seq
.......................................................................uaucCcagcccgcuuuguugacu.................   25     1   seq
.......................................................................uaucacagcccgcuuuNuugacu.................   1      1   seq
.......................................................................uaucacUgcccgcuuuguugacu.................   15     1   seq
.......................................................................Caucacagcccgcuuuguugacu.................   134    1   seq
.......................................................................uaucacagcccgcuuuguugacG.................   2102   1   seq
.......................................................................uaucacagcccgcuGuguugacu.................   41     1   seq
.......................................................................uNucacagcccgcuuuguugacu.................   25     1   seq
.......................................................................uaucacagcccgcGuuguugacu.................   43     1   seq
.......................................................................uaucacagcccgcuuugAugacu.................   350    1   seq
.......................................................................uaGcacagcccgcuuuguugacu.................   9      1   seq
.......................................................................uaucacagcccgcuAuguugacu.................   308    1   seq
.......................................................................uUucacagcccgcuuuguugacu.................   77     1   seq
.......................................................................uaAcacagcccgcuuuguugacu.................   1018   1   seq
.......................................................................uaucacagcccgcuuuguugacuU................   921    1   seq
.......................................................................uaucacagcccgcuuuguugacuA................   149    1   seq
.......................................................................uaucacagcccgcuuuguugacuc................   46     0   seq
.......................................................................uaucacagcccgcuuuguugacuG................   7      1   seq
.......................................................................uaucacagcccgcuuuguugacuN................   1      1   seq
.......................................................................uaucacagcccgcuuuguugacucu...............   3      0   seq
.......................................................................uaucacagcccgcuuuguugacucA...............   2      1   seq
.......................................................................uaucacagcccgcuuuguugacuAu...............   18     1   seq
.......................................................................uaucacagcccgUuuuguugacucu...............   1      1   seq
.......................................................................uaucacagcccgcuuuguugacuUu...............   158    1   seq
........................................................................aucacagcccgcuuuguugac..................   8      0   seq
........................................................................aucacagcccgcuuuguugacu.................   198    0   seq
........................................................................aucacagcccgcuuugAugacu.................   1      1   seq
........................................................................Uucacagcccgcuuuguugacu.................   2      1   seq
........................................................................aucacagcccgcuuuguugacG.................   1      1   seq
```

```
                 Star                                                 Mature
gaucaaucauggaggaaccgacaaaguggcugcgauguguucucuuccuccuccgauaugcgucgcaccauaucacagcccgcuuuguugacucugccgugcaauacucu
.......................................................................aucacagcccgcuuuguugacuA................   1   1   seq
.......................................................................aucacagcccgcuuuguugacuc................   4   0   seq
........................................................................ucacagcccgcuuuguugac..................   1   0   seq
........................................................................ucacagcccgcuuuguugacu.................   18  0   seq
.........................................................................Uacagcccgcuuuguugac..................   1   1   seq
.........................................................................cacagcccgcuuuguugacu.................   3   0   seq
..........................................................................acagcccgcuuuguugac..................   4   0   seq
..........................................................................acagcccgcuuuguugacA.................   2   1   seq
..........................................................................acagcccgcuuuguugacu.................   55  0   seq
..........................................................................acagcccgcAuuguugacu.................   1   1   seq
...........................................................................cagcccgcuuuguugacu.................   14  0   seq
```
